# Renal Angiomyolipoma Rupture Following COVID-19 Infection: A Case Report

**DOI:** 10.7759/cureus.41734

**Published:** 2023-07-11

**Authors:** Mohammed Abdurabu, Akram Al-warqi, Ebrahim M Ebrahim, Salman Mirza, Jouhar Kolleri

**Affiliations:** 1 Emergency Medicine, Hamad Medical Corporation, Doha, QAT; 2 Radiology, Hamad Medical Corporation, Doha, QAT; 3 Family Medicine, Medical Education, Hamad Medical Corporation, Doha, QAT; 4 Clinical Imaging, Hamad Medical Corporation, Doha, QAT

**Keywords:** infection, case report, rupture, renal angiomyolipoma, covid – 19

## Abstract

The novel Coronavirus (COVID-19) is one of the most recent Pandemics that invaded the earth and is still active. It caused and is still causing hundreds of thousands of patients high morbidity and mortality rates, with no definitive cure at this moment. COVID-19 has been proven to be associated with pathologic changes in coagulation, characterized by either thromboembolic or bleeding events. We describe this case of a 44-year-old male patient who walked into our emergency department with flank pain and was later discovered to have had renal angiomyolipoma (AML) rupture during his COVID-19 infection, ultimately requiring admission for hemorrhage control via interventional radiology (IR) drainage. Here, we discuss the role of front-line physicians and how they should keep a low threshold for the different presentations that could be associated with COVID-19 infection, such as what was found in this case.

## Introduction

Renal angiomyolipoma (AML) is an uncommon benign tumor of the kidneys. Histopathologically, it consists of blood vessels, smooth muscle, and adipose tissue [1]. Along with other tumors, renal AML is referred to as a neoplasm with perivascular epithelioid differentiation (1). AML occurs either sporadically or in association with tuberous sclerosis complex (TSC) or pulmonary lymphangioleiomyomatosis (LAM). It usually occurs in middle-aged patients and is more frequent in females than males (2:1) [2,3]. Despite the benign course of the disease and the fact that most patients are asymptomatic with normal kidney function, some patients present with flank pain, hematuria, or retroperitoneal hemorrhage. With advances in body imaging, incidental detection of these tumors is responsible for 80% of the cases [4]. As suggested by ultrasonography, renal AML is confirmed with computed tomography (CT) or magnetic resonance imaging (MRI). Rupture of renal AML with hemorrhage is reported in 15% of patients (4). With the recent pandemic of the novel Coronavirus (COVID-19), various thromboembolic and bleeding events have been reported with possible associations [5-8]. Our report describes a 44-year-old male COVID-19 patient with ruptured renal AML.

## Case presentation

A 44-year-old gentleman presented to the emergency department with complaints of sudden onset of left flank pain lasting four hours. The pain was progressive in nature, radiating to the back with a severity of 10/10. There were no exacerbating factors for the pain, and it was not relieved by analgesia. The patient also complained of symptoms of vomiting, coughing, and an upper respiratory tract infection. No history of dysuria, hematuria, irritative urinary symptoms like frequency, urgency, or nocturia, no obstructive urinary symptoms, and no sensation of incomplete emptying of the bladder were noted. He is not a stone former. There have been no similar illnesses in the past and no relevant family history. His vitals were stable. On examination, the abdomen was soft and lax. Severe left flank tenderness was noted. Laboratory examination revealed a positive COVID polymerase chain reaction (PCR) with a cycle threshold of 20.7.

**Figure 1:**
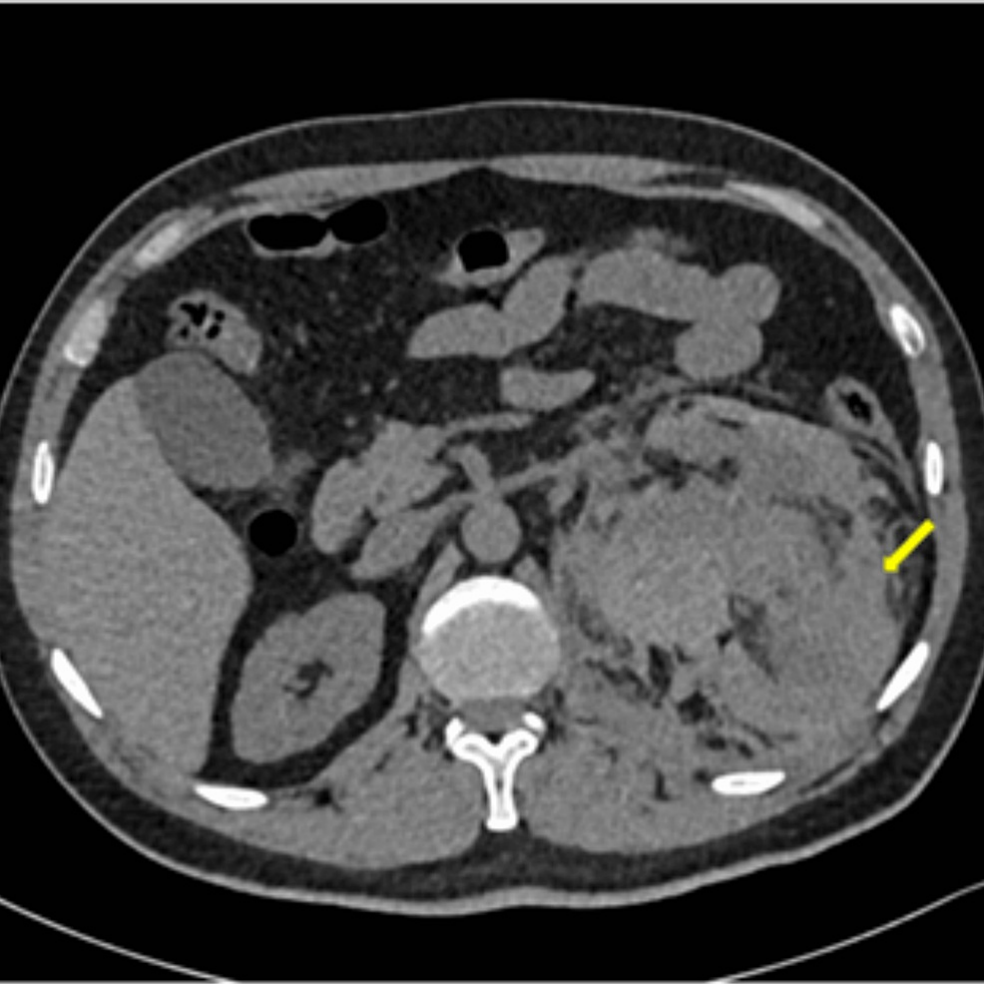
CT urinary tract: axial cuts showing large retroperitoneal hematoma in the left perinephric space extending caudally to the left lower abdomen (yellow arrows).

A CT of the urinary tract was done, which demonstrated a large retroperitoneal hematoma in the left perinephric space that extended caudally to the left lower abdomen. No renal calculus or evidence of obstructive uropathy was seen. The right kidney appeared unremarkable. The rest of the upper abdominal solid organs appeared within normal limits. These findings were suggestive of a large left perinephric hematoma (Figure 1).

**Figure 2:**
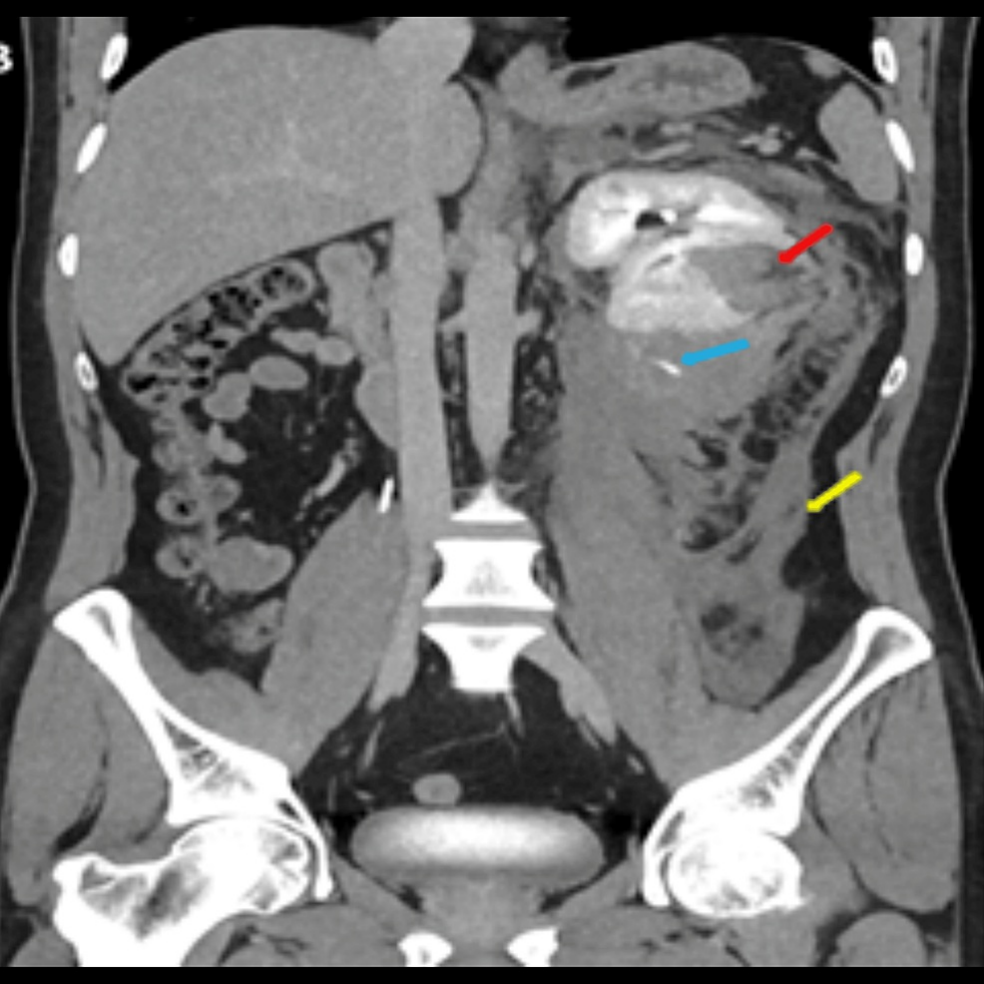
CT abdomen with contrast: coronal cuts showing a large left perinephric hematoma extending inferiorly in the retroperitoneum, reaching the left iliac fossa (yellow arrows). An ill-defined partially exophytic heterogeneously enhancing lesion was noted at the interpolar area of the left kidney (red arrows) with a prominent vessel at the periphery of the lesion, suspicious of minimal contrast extravasation (blue arrow).

An abdominal CT scan with contrast was done to further evaluate the lesion, which showed a sizable left perinephric hematoma extending inferiorly in the retroperitoneum, reaching the left iliac fossa. The hematoma was displacing the left kidney anteriorly and compressing the renal parenchyma, suggesting a page kidney. An ill-defined, partially exophytic, heterogeneously enhancing lesion was noted at the interpolar region of the left kidney, approximately 45 mm × 45 mm × 40 mm. Multiple prominent vessels were seen at the periphery of the lesion, raising suspicion of minimal contrast extravasation at the site of the lesion. A prominent vessel was seen adjacent to the inferior pole of the left kidney, with active IV contrast extravasation noted in the delayed study. No sizable radio-dense urinary calculi or hydronephrosis were seen. No pneumoperitoneum or other obvious acute abnormalities were noted in the abdomen and pelvis. These findings gave the impression of a ruptured angiomyolipoma (Figure 2).

**Figure 3:**
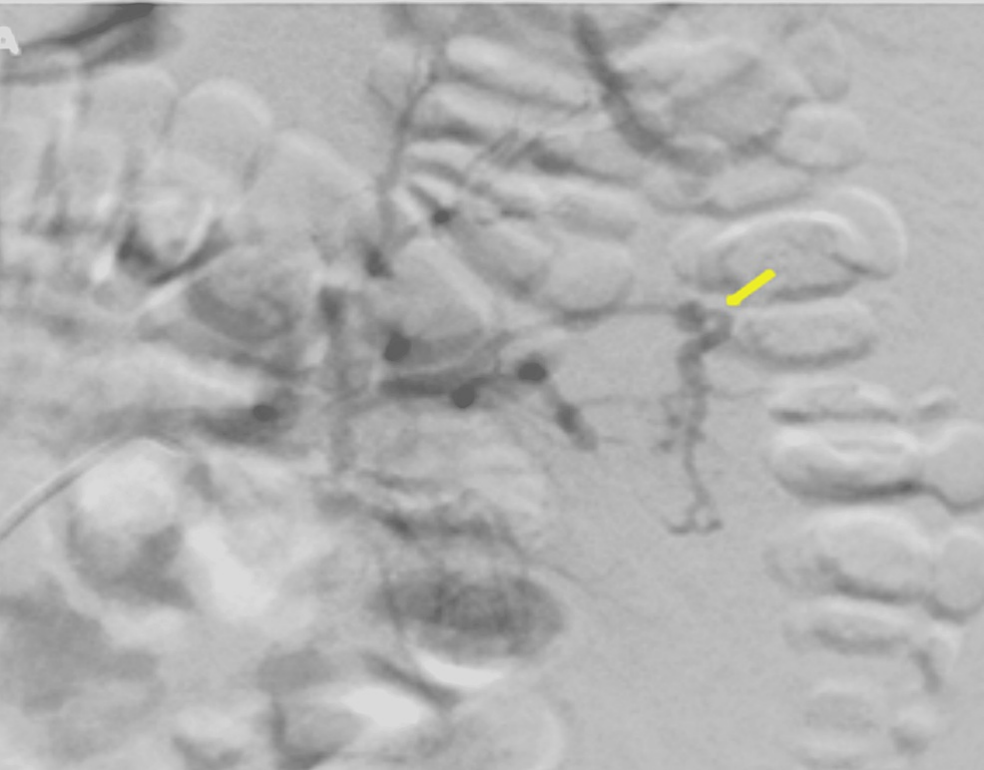
Selective digital subtraction angiography showing abnormal vascular structure in the mid pole of the left kidney with no extravasation (yellow arrow).

**Figure 4:**
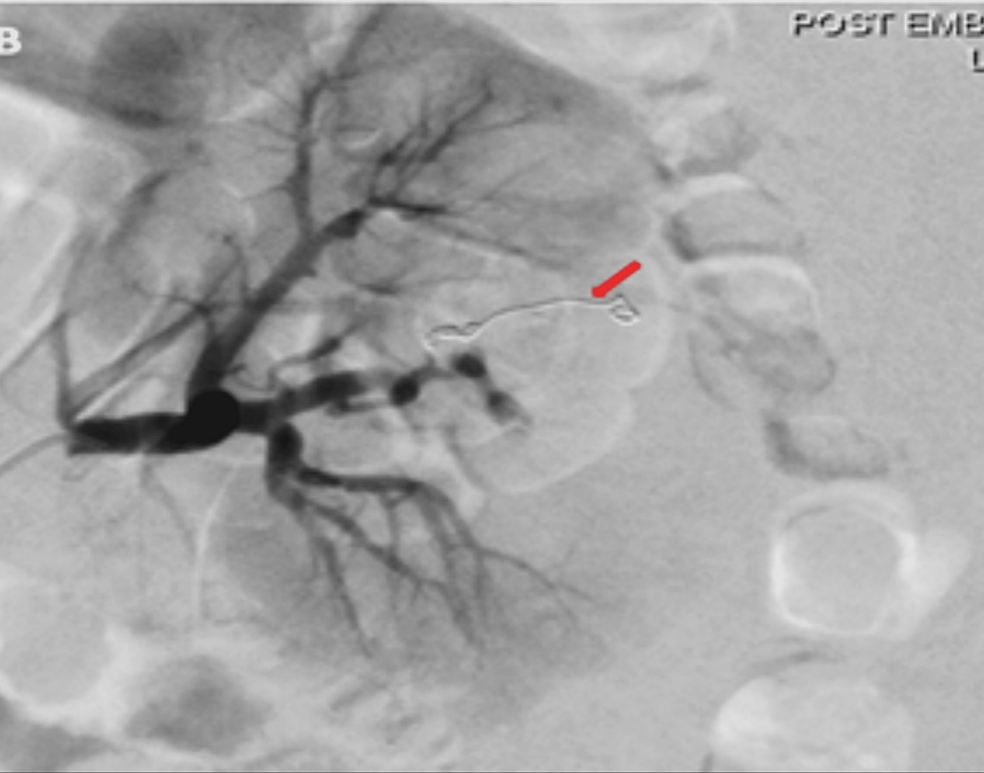
Selective digital subtraction angiography which showed complete occlusion of the feeding vessel post embolization (red arrow).

The patient was kept at nil per mouth and started on intravenous ceftriaxone, 2 g daily for seven days, and intravenous fluids. The patient was posted for left renal angioembolization by the interventional radiology (IR) team. Selective digital subtraction angiography (DSA) showed highly abnormal vascular structures in the mid-pole of the left kidney with no extravasation. The feeding vessel was closed with a vortex coil, and gel foam was used to close the remaining feeding vessels. At the end of the procedure, DSA showed complete occlusion of the feeding vessels (Figures 3-4).

**Figure 5:**
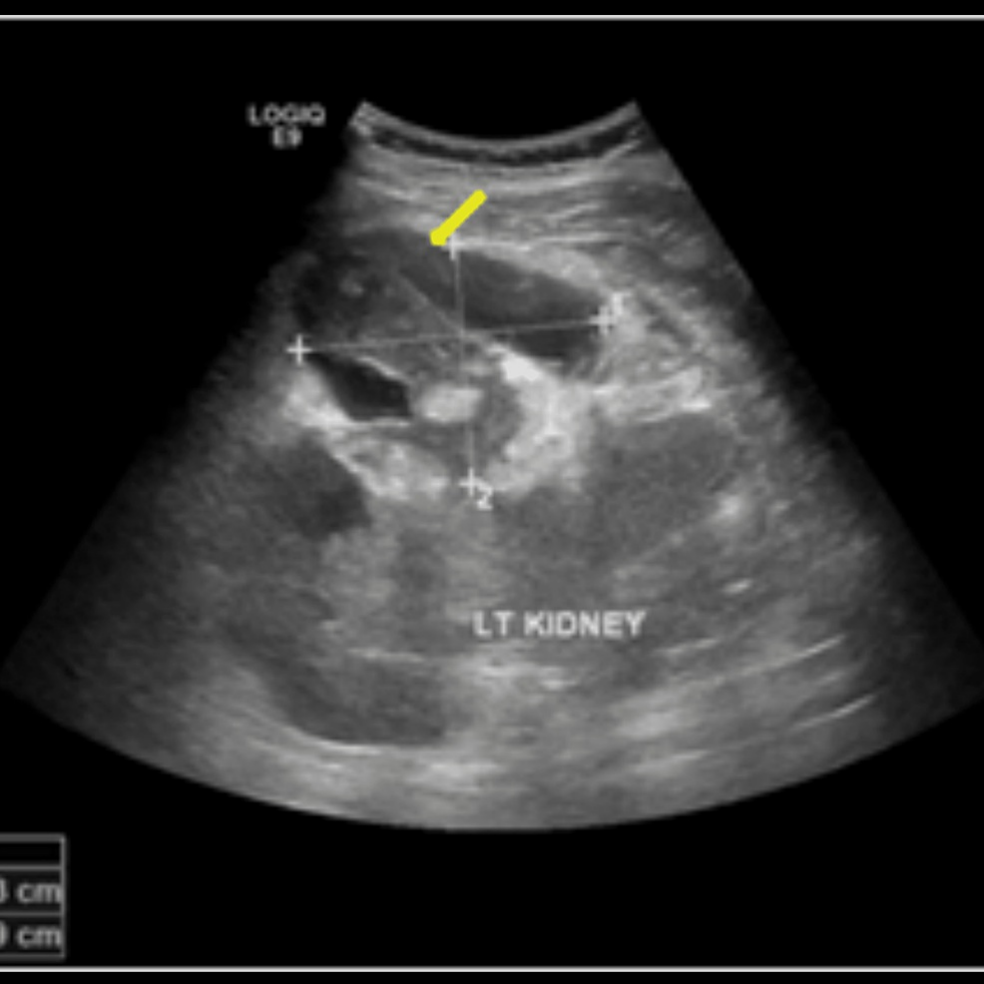
Ultrasound urinary tract showing a heterogenous cortical lesion at the interpolar region of the left kidney (yellow arrow).

**Figure 6:**
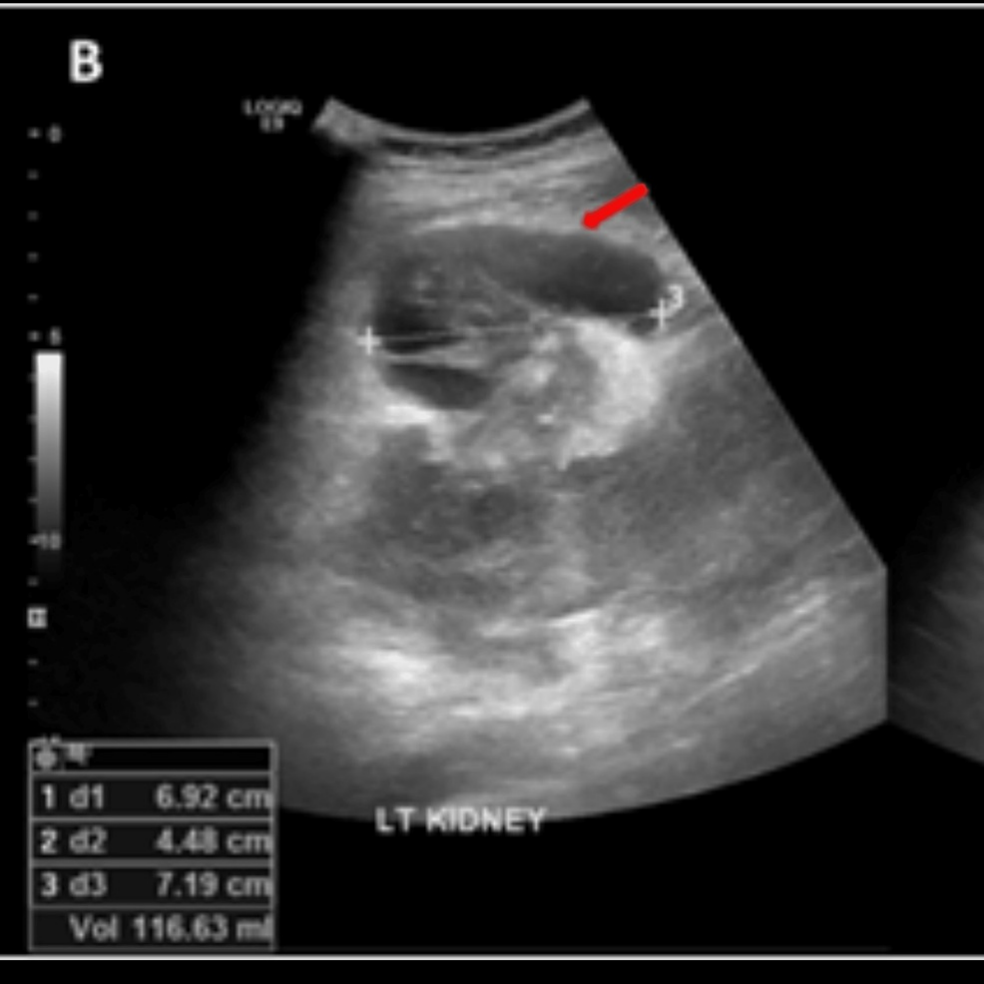
Ultrasound urinary tract showing perinephric hematoma (red arrows).

Post-procedural days were unremarkable, and he tested negative on the 10th day of admission. An ultrasound of the urinary tract was done, which showed an ill-defined left renal outline with a 7 cm × 5 cm heterogenous cortical lesion at the interpolar region. A perinephric hematoma measuring 116 mL was also noted (Figures 5-6).

The hypervascular structure needs to be investigated later to exclude oncocytoma, and he was advised to follow up after six weeks with an MRI.

## Discussion

Angiomyolipomas are benign mesenchymal lesions of the kidney comprised of mature adipose tissue, dysmorphic blood vessels, and smooth muscle. As with other renal tumors, the increased use of cross-sectional abdominal imaging has led to a rise in the diagnosis of these lesions. About 80% of AMLs are sporadic and not associated with any genetic syndrome. The remaining cases are associated with TSC and sporadic LAM [9]. Renal AMLs larger than 4 cm have a significantly higher risk of rupture; however, rupture can occur at smaller sizes, and larger AMLs may remain stable. Other risk factors for rupture are pregnancy and aneurysms [7]. In our case, the patient had a large 5 cm × 7 cm angiomyolipoma, which posed an increased risk of rupture in addition to his COVID-19 infection.

The patient initially presented with flank pain that was out of proportion and not relieved with analgesia, which is the usual presenting complaint [10]. A CT scan showed a retroperitoneal hematoma and a 5 × 7 mass in the left kidney with signs consistent with ruptured angiomyolipoma [7,10,11].

Despite the difficulty in establishing causality between COVID-19 infection and renal AML rupture, there have been many reports linking the two incidents. The only other risk factor in our patient was the size of the AML. Other spontaneous bleeding complications have been described after infection with COVID-19, such as retroperitoneal hematoma, gastrointestinal bleeding, hemopneumothorax, and cerebral hemorrhage [5].

## Conclusions

COVID-19 can participate in bleeding as well as thrombosis. It has been proven that COVID-19 is associated with higher rates of thrombosis and bleeding in hospitalized patients with similar illnesses. This dysregulated coagulation is suggested to be due to endothelial disruption and autoimmunity induced by the coronavirus infection. Therefore, we feel that this case is worth reporting given the fact that COVID-19 was an important contributor to this patient’s AML rupture.
